# Tris(diphenyl­propyl­phosphine-κ*P*)-μ_2_-iodido-tri-μ_3_-sulfido-sulfidotricopper(I)tungsten(VI)

**DOI:** 10.1107/S1600536808030390

**Published:** 2008-10-18

**Authors:** Guodong Tang

**Affiliations:** a Huaiyin Teachers College, Huai’an 223001, Jiangsu, People’s Republic of China

## Abstract

A neutral W/S/Cu cluster, [Cu_3_WIS_4_(C_15_H_17_P)_3_], was formed by the reaction of tetra­thio­tungstate(VI), CuI and diphenyl­propyl­phosphine (dpp) in dimethyl­formamide. The title compound exhibits a neutral half-open cubane-like skeleton, with Cu—I bonds of 2.8056 (8) and 2.9008 (8) Å, and one Cu⋯I short contact of 3.1722 (6) Å. The W atom exhibits a tetra­hedral coordination geometry through bonding to three μ_3_-S and one terminal S atom. The three Cu^I^ atoms are in two different coordination environments: one Cu atom exhibits a triangular coordination geometry being coordinated by one P atom from dpp and two μ_3_-S atoms, whereas the remaining two Cu centers are tetra­hedrally coordinated, forming the CuPIS_2_ core.

## Related literature

For an anionic W/S/Cu cluster with a half-open cubane-like skeleton, see: Hou, Liang *et al.* (1996 ▶). Mo(W)/S/Cu(Ag) clusters have been reviewed by Hou, Xin *et al.* (1996 ▶) and Niu *et al.* (2004 ▶). The potential applications of Mo(W)/S/Cu(Ag) clusters have been reviewed by Müller *et al.* (1981 ▶) and Zhang *et al.* (2007 ▶).
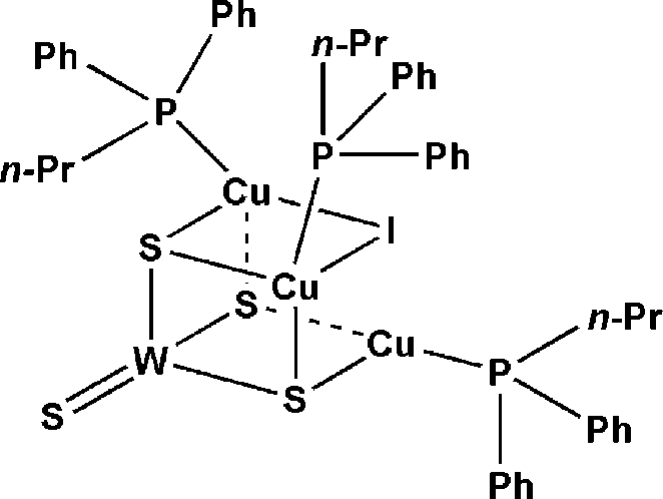


## Experimental

### 

#### Crystal data

[Cu_3_WIS_4_(C_15_H_17_P)_3_]
*M* *_r_* = 1314.38
Triclinic, 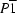

*a* = 11.7161 (10) Å
*b* = 13.1040 (13) Å
*c* = 17.1958 (15) Å
α = 86.491 (6)°
β = 77.568 (5)°
γ = 74.103 (5)°
*V* = 2479.5 (4) Å^3^
*Z* = 2
Mo *K*α radiation
μ = 4.50 mm^−1^
*T* = 193 (2) K
0.38 × 0.37 × 0.13 mm

#### Data collection

Rigaku Mercury diffractometer
Absorption correction: multi-scan (*SADABS*; Sheldrick, 1996 ▶) *T*_min_ = 0.196, *T*_max_ = 0.557
24291 measured reflections
9073 independent reflections
7765 reflections with *I* > 2σ(*I*)
*R*_int_ = 0.039

#### Refinement

*R*[*F* ^2^ > 2σ(*F* ^2^)] = 0.039
*wR*(*F* ^2^) = 0.083
*S* = 1.09
9073 reflections
506 parameters
H-atom parameters constrained
Δρ_max_ = 1.36 e Å^−3^
Δρ_min_ = −1.20 e Å^−3^

### 

Data collection: *CrystalClear* (Rigaku, 2000 ▶); cell refinement: *CrystalClear*; data reduction: *CrystalStructure* (Rigaku, 2000 ▶); program(s) used to solve structure: *SHELXTL* (Sheldrick, 2008 ▶); program(s) used to refine structure: *SHELXTL*; molecular graphics: *SHELXTL*; software used to prepare material for publication: *SHELXTL*.

## Supplementary Material

Crystal structure: contains datablocks I, global. DOI: 10.1107/S1600536808030390/gk2166sup1.cif

Structure factors: contains datablocks I. DOI: 10.1107/S1600536808030390/gk2166Isup2.hkl

Additional supplementary materials:  crystallographic information; 3D view; checkCIF report

## Figures and Tables

**Table 1:** Selected bond lengths (Å)

W1—S4	2.1314 (15)
W1—S1	2.2445 (13)
W1—S3	2.2483 (13)
W1—S2	2.2533 (13)
W1—Cu3	2.7033 (7)
W1—Cu1	2.7173 (8)
W1—Cu2	2.7272 (7)
Cu1—P1	2.2150 (16)
Cu1—S3	2.3094 (15)
Cu1—S1	2.3194 (14)
Cu2—P2	2.2241 (14)
Cu2—S1	2.3015 (15)
Cu2—S2	2.3036 (15)
Cu3—P3	2.2209 (15)
Cu3—S2	2.2833 (15)
Cu3—S3	2.2849 (15)

**Fig. 1.**
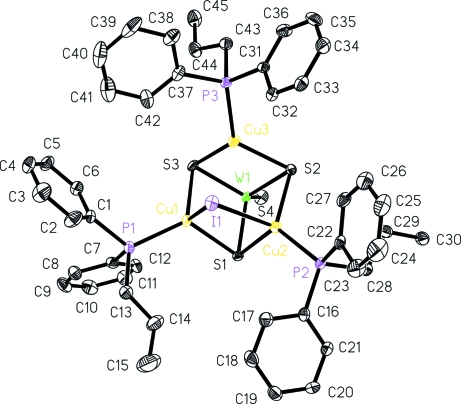
The molecular structure of the title compound, with atom labels and 30% probability displacement ellipsoids. All H atoms have been omitted.

## supplementary crystallographic information

### Comment

Mo(W)/S/Cu(Ag) clusters have attracted much attention for their diverse architectures (How, Xin *et al.*, 1996; Niu *et al.*, 2004) and potential applications (Müller *et al.*, 1981; Zhang *et al.*, 2007) but the crystal structures of these clusters containing diphenylpropylphosphine ligands have not been reported until now. In order to explore the chemistry of Mo(W)/S/Cu(Ag) clusters extensively,we have synthesized the cluster by reaction in solution at normal temperatures.

As illustrated in Fig. 1, the title compound has a half-open cubane-like skeleton, in which W atom adopts a distorted tetrahedral coordination geometry through bonding to three µ_3_-S and a terminal S atoms. Three Cu atoms have two kinds of coordination environments: one is coordinated by one P atom from dpp and two µ_3_-S forming a triangular coordination geometry, and another two have a tetrahedral coordination geometry formed by one P atom from dpp, one µ_2_-I and two µ_3_-S. Interestingly, the Cu—I bonds  [2.8056 (8) Å and 2.9008 (8) Å] in the title compound are obviously shorter than those [(2.942 (4) Å] in theanionic W/S/Cu cluster with the same skeleton (Hou, Liang *et al.*, 1996).

### Experimental

3 mmol CuI, 1 mmol [NH_4_]_2_WS_4_ and 3 mmol dpp were added to 5 mL dmf with thorough stirring for 5 minutes. After filtration, the orange-red filtrate was carefully laid on the surface with 30 ml *i*-PrOH. Yellow block crystals were obtained after ten days. Yield: 0.0341 g in pure form, 70.1% (based on W). Analysis calculated for C_45_H_51_Cu_3_IP_3_S_4_W: C 41.12, H 3.91%; found: C 41.10, H 3.90%. IR: ν, cm^-1^,480.68, 444.74 s (W-µ_3_-S).

### Refinement

H atoms were positioned geometrically with C-H = 0.95-0.99 Å and refined in riding model approximation, with *U*_iso_ = 1.5*U*_eq_ for methyl H atoms and 1.2*U*_eq_ for H atoms from methylene and phenyl groups.

### Crystal data

**Table:**

[Cu_3_WIS_4_(C_15_H_17_P)_3_]	*Z* = 2
*M**_r_* = 1314.38	*F*(000) = 1288
Triclinic, *P*1	*D*_x_ = 1.760 Mg m^−^^3^
Hall symbol: -P 1	Mo *K*α radiation, λ = 0.71070 Å
*a* = 11.7161 (10) Å	Cell parameters from 9051 reflections
*b* = 13.1040 (13) Å	θ = 3.2–25.3°
*c* = 17.1958 (15) Å	µ = 4.50 mm^−^^1^
α = 86.491 (6)°	*T* = 193 K
β = 77.568 (5)°	Block, orange
γ = 74.103 (5)°	0.38 × 0.37 × 0.13 mm
*V* = 2479.5 (4) Å^3^	

### Data collection

**Table:**

Rigaku Mercury diffractometer	9073 independent reflections
Radiation source: fine-focus sealed tube	7765 reflections with *I* > 2σ(*I*)
graphite	*R*_int_ = 0.039
Detector resolution: 7.31 pixels mm^-1^	θ_max_ = 25.4°, θ_min_ = 3.2°
ω scans	*h* = −14→13
Absorption correction: multi-scan (SADABS; Sheldrick, 1996)	*k* = −15→15
*T*_min_ = 0.196, *T*_max_ = 0.557	*l* = −20→18
24291 measured reflections	

### Refinement

**Table:**

Refinement on *F*^2^	Primary atom site location: structure-invariant direct methods
Least-squares matrix: full	Secondary atom site location: difference Fourier map
*R*[*F*^2^ > 2σ(*F*^2^)] = 0.039	Hydrogen site location: inferred from neighbouring sites
*wR*(*F*^2^) = 0.083	H-atom parameters constrained
*S* = 1.09	*w* = 1/[σ^2^(*F*_o_^2^) + (0.0305*P*)^2^ + 3.084*P*] where *P* = (*F*_o_^2^ + 2*F*_c_^2^)/3
9073 reflections	(Δ/σ)_max_ = 0.001
506 parameters	Δρ_max_ = 1.36 e Å^−^^3^
0 restraints	Δρ_min_ = −1.20 e Å^−^^3^

### Special details

**Table:**

Geometry. All e.s.d.'s (except the e.s.d. in the dihedral angle between two l.s. planes) are estimated using the full covariance matrix. The cell e.s.d.'s are taken into account individually in the estimation of e.s.d.'s in distances, angles and torsion angles; correlations between e.s.d.'s in cell parameters are only used when they are defined by crystal symmetry. An approximate (isotropic) treatment of cell e.s.d.'s is used for estimating e.s.d.'s involving l.s. planes.
Refinement. Refinement of *F*^2^ against ALL reflections. The weighted *R*-factor *wR* and goodness of fit *S* are based on *F*^2^, conventional *R*-factors *R* are based on *F*, with *F* set to zero for negative *F*^2^. The threshold expression of *F*^2^ > σ(*F*^2^) is used only for calculating *R*-factors(gt) *etc*. and is not relevant to the choice of reflections for refinement. *R*-factors based on *F*^2^ are statistically about twice as large as those based on *F*, and *R*- factors based on ALL data will be even larger.

### Fractional atomic coordinates and isotropic or equivalent isotropic displacement parameters (Å^2^)

**Table:**

	*x*	*y*	*z*	*U*_iso_*/*U*_eq_	
W1	0.231242 (18)	0.395552 (17)	0.228219 (12)	0.02162 (7)	
I1	0.04088 (3)	0.15726 (3)	0.25752 (2)	0.03494 (11)	
Cu1	0.27335 (6)	0.18233 (5)	0.24969 (4)	0.03150 (17)	
Cu2	0.03224 (6)	0.35612 (6)	0.32704 (4)	0.02940 (17)	
Cu3	0.11527 (6)	0.32641 (6)	0.13088 (4)	0.03415 (18)	
S1	0.22840 (12)	0.31168 (11)	0.34612 (7)	0.0243 (3)	
S2	0.03705 (12)	0.46591 (11)	0.21720 (8)	0.0270 (3)	
S3	0.31753 (12)	0.27256 (11)	0.13230 (8)	0.0280 (3)	
S4	0.32984 (14)	0.51184 (13)	0.21978 (9)	0.0412 (4)	
P1	0.39067 (13)	0.02190 (11)	0.26649 (9)	0.0293 (3)	
P2	−0.12091 (12)	0.39157 (11)	0.43205 (8)	0.0235 (3)	
P3	0.04133 (13)	0.30253 (11)	0.02686 (8)	0.0266 (3)	
C1	0.4102 (5)	−0.0724 (4)	0.1889 (3)	0.0290 (13)	
C2	0.3512 (6)	−0.1520 (5)	0.1976 (4)	0.0519 (19)	
H2	0.2976	−0.1594	0.2463	0.062*	
C3	0.3705 (8)	−0.2213 (6)	0.1347 (4)	0.065 (2)	
H3	0.3294	−0.2755	0.1409	0.078*	
C4	0.4476 (7)	−0.2122 (5)	0.0642 (4)	0.0519 (18)	
H4	0.4610	−0.2605	0.0221	0.062*	
C5	0.5052 (6)	−0.1336 (5)	0.0547 (4)	0.0462 (17)	
H5	0.5581	−0.1267	0.0055	0.055*	
C6	0.4871 (5)	−0.0641 (5)	0.1159 (3)	0.0378 (15)	
H6	0.5277	−0.0095	0.1082	0.045*	
C7	0.5437 (5)	0.0212 (5)	0.2712 (3)	0.0326 (13)	
C8	0.6366 (5)	−0.0748 (5)	0.2656 (4)	0.0400 (15)	
H8	0.6197	−0.1396	0.2570	0.048*	
C9	0.7516 (6)	−0.0749 (6)	0.2725 (4)	0.0492 (17)	
H9	0.8142	−0.1394	0.2680	0.059*	
C10	0.7756 (6)	0.0192 (6)	0.2860 (4)	0.0501 (18)	
H10	0.8547	0.0187	0.2916	0.060*	
C11	0.6864 (6)	0.1135 (6)	0.2916 (4)	0.0490 (17)	
H11	0.7037	0.1782	0.3003	0.059*	
C12	0.5709 (6)	0.1129 (5)	0.2844 (4)	0.0390 (15)	
H12	0.5090	0.1779	0.2889	0.047*	
C13	0.3401 (8)	−0.0445 (6)	0.3588 (4)	0.0693 (14)	
H13A	0.2615	−0.0582	0.3577	0.083*	
H13B	0.3997	−0.1138	0.3622	0.083*	
C14	0.3255 (8)	0.0205 (6)	0.4322 (4)	0.0693 (14)	
H14A	0.3979	0.0476	0.4269	0.083*	
H14B	0.2543	0.0827	0.4340	0.083*	
C15	0.3100 (8)	−0.0366 (6)	0.5076 (4)	0.0693 (14)	
H15A	0.3786	−0.0998	0.5060	0.104*	
H15B	0.2344	−0.0581	0.5161	0.104*	
H15C	0.3066	0.0096	0.5512	0.104*	
C16	−0.0945 (5)	0.3076 (4)	0.5184 (3)	0.0270 (12)	
C17	−0.0496 (6)	0.1991 (5)	0.5066 (4)	0.0390 (15)	
H17	−0.0319	0.1712	0.4544	0.047*	
C18	−0.0303 (6)	0.1313 (5)	0.5699 (4)	0.0449 (16)	
H18	−0.0006	0.0570	0.5609	0.054*	
C19	−0.0537 (5)	0.1701 (5)	0.6459 (4)	0.0407 (15)	
H19	−0.0398	0.1231	0.6893	0.049*	
C20	−0.0970 (6)	0.2770 (5)	0.6583 (3)	0.0440 (16)	
H20	−0.1133	0.3042	0.7107	0.053*	
C21	−0.1173 (6)	0.3462 (5)	0.5954 (3)	0.0397 (15)	
H21	−0.1470	0.4203	0.6050	0.048*	
C22	−0.2653 (5)	0.3793 (4)	0.4156 (3)	0.0279 (13)	
C23	−0.3520 (6)	0.3547 (6)	0.4772 (4)	0.0503 (18)	
H23	−0.3335	0.3353	0.5282	0.060*	
C24	−0.4655 (6)	0.3587 (7)	0.4637 (5)	0.066 (2)	
H24	−0.5241	0.3400	0.5054	0.080*	
C25	−0.4946 (6)	0.3891 (6)	0.3913 (5)	0.058 (2)	
H25	−0.5736	0.3939	0.3832	0.070*	
C26	−0.4092 (6)	0.4123 (6)	0.3311 (4)	0.0530 (19)	
H26	−0.4288	0.4323	0.2806	0.064*	
C27	−0.2940 (5)	0.4073 (5)	0.3421 (4)	0.0383 (15)	
H27	−0.2352	0.4230	0.2993	0.046*	
C28	−0.1643 (5)	0.5282 (4)	0.4691 (3)	0.0262 (12)	
H28A	−0.2342	0.5365	0.5145	0.031*	
H28B	−0.0960	0.5404	0.4890	0.031*	
C29	−0.1980 (5)	0.6121 (4)	0.4067 (3)	0.0345 (14)	
H29A	−0.1282	0.6045	0.3612	0.041*	
H29B	−0.2666	0.6005	0.3867	0.041*	
C30	−0.2334 (6)	0.7239 (4)	0.4396 (3)	0.0384 (15)	
H30A	−0.3011	0.7314	0.4856	0.058*	
H30B	−0.2580	0.7751	0.3983	0.058*	
H30C	−0.1639	0.7375	0.4561	0.058*	
C31	−0.1234 (5)	0.3445 (4)	0.0415 (3)	0.0257 (12)	
C32	−0.1901 (5)	0.3094 (4)	0.1096 (3)	0.0322 (13)	
H32	−0.1495	0.2679	0.1478	0.039*	
C33	−0.3154 (5)	0.3345 (5)	0.1222 (3)	0.0364 (14)	
H33	−0.3598	0.3074	0.1678	0.044*	
C34	−0.3758 (5)	0.3977 (5)	0.0697 (4)	0.0432 (16)	
H34	−0.4618	0.4159	0.0793	0.052*	
C35	−0.3106 (6)	0.4350 (6)	0.0024 (4)	0.0500 (18)	
H35	−0.3520	0.4795	−0.0342	0.060*	
C36	−0.1849 (5)	0.4074 (5)	−0.0117 (3)	0.0389 (15)	
H36	−0.1407	0.4322	−0.0585	0.047*	
C37	0.0811 (5)	0.1674 (5)	−0.0073 (3)	0.0334 (14)	
C38	0.0241 (6)	0.1409 (5)	−0.0636 (4)	0.0476 (17)	
H38	−0.0421	0.1916	−0.0793	0.057*	
C39	0.0649 (8)	0.0396 (6)	−0.0965 (4)	0.069 (3)	
H39	0.0272	0.0207	−0.1350	0.082*	
C40	0.1613 (8)	−0.0331 (6)	−0.0723 (6)	0.073 (3)	
H40	0.1900	−0.1020	−0.0952	0.087*	
C41	0.2148 (7)	−0.0084 (6)	−0.0176 (6)	0.072 (2)	
H41	0.2803	−0.0598	−0.0017	0.086*	
C42	0.1754 (6)	0.0913 (5)	0.0161 (4)	0.0480 (17)	
H42	0.2132	0.1078	0.0556	0.058*	
C43	0.0976 (5)	0.3685 (5)	−0.0647 (3)	0.0347 (14)	
H43A	0.0603	0.3537	−0.1077	0.042*	
H43B	0.0725	0.4461	−0.0560	0.042*	
C44	0.2350 (5)	0.3321 (5)	−0.0910 (4)	0.0432 (16)	
H44A	0.2723	0.3505	−0.0492	0.052*	
H44B	0.2605	0.2540	−0.0968	0.052*	
C45	0.2813 (6)	0.3820 (5)	−0.1691 (4)	0.0529 (19)	
H45A	0.2459	0.3630	−0.2110	0.079*	
H45B	0.3699	0.3560	−0.1834	0.079*	
H45C	0.2581	0.4594	−0.1634	0.079*	

### Atomic displacement parameters (Å^2^)

**Table:**

	*U*^11^	*U*^22^	*U*^33^	*U*^12^	*U*^13^	*U*^23^
W1	0.01802 (12)	0.02671 (13)	0.01979 (12)	−0.00629 (9)	−0.00151 (9)	−0.00471 (9)
I1	0.0288 (2)	0.0363 (2)	0.0431 (2)	−0.01358 (18)	−0.00673 (17)	−0.00604 (18)
Cu1	0.0310 (4)	0.0281 (4)	0.0307 (4)	0.0010 (3)	−0.0059 (3)	−0.0068 (3)
Cu2	0.0182 (3)	0.0405 (4)	0.0250 (4)	−0.0046 (3)	0.0008 (3)	0.0003 (3)
Cu3	0.0281 (4)	0.0449 (4)	0.0304 (4)	−0.0048 (3)	−0.0112 (3)	−0.0121 (3)
S1	0.0218 (7)	0.0306 (8)	0.0204 (7)	−0.0050 (6)	−0.0057 (5)	−0.0032 (6)
S2	0.0207 (7)	0.0312 (8)	0.0257 (7)	−0.0008 (6)	−0.0054 (6)	−0.0007 (6)
S3	0.0226 (7)	0.0374 (8)	0.0210 (7)	−0.0046 (6)	−0.0002 (6)	−0.0096 (6)
S4	0.0408 (9)	0.0425 (10)	0.0435 (9)	−0.0205 (8)	−0.0023 (7)	−0.0038 (7)
P1	0.0255 (8)	0.0270 (8)	0.0332 (8)	−0.0029 (6)	−0.0053 (6)	−0.0056 (6)
P2	0.0173 (7)	0.0291 (8)	0.0242 (7)	−0.0073 (6)	−0.0020 (6)	−0.0035 (6)
P3	0.0241 (8)	0.0305 (8)	0.0261 (8)	−0.0056 (6)	−0.0076 (6)	−0.0065 (6)
C1	0.030 (3)	0.022 (3)	0.033 (3)	−0.004 (2)	−0.004 (3)	−0.006 (2)
C2	0.067 (5)	0.056 (4)	0.037 (4)	−0.034 (4)	0.005 (3)	−0.007 (3)
C3	0.100 (6)	0.046 (4)	0.061 (5)	−0.046 (4)	−0.006 (5)	−0.004 (4)
C4	0.066 (5)	0.037 (4)	0.051 (4)	−0.006 (4)	−0.013 (4)	−0.018 (3)
C5	0.041 (4)	0.056 (4)	0.035 (4)	−0.003 (3)	0.000 (3)	−0.015 (3)
C6	0.036 (4)	0.037 (4)	0.037 (4)	−0.008 (3)	−0.003 (3)	−0.004 (3)
C7	0.035 (3)	0.035 (3)	0.032 (3)	−0.011 (3)	−0.013 (3)	0.002 (3)
C8	0.036 (4)	0.039 (4)	0.046 (4)	−0.010 (3)	−0.011 (3)	0.003 (3)
C9	0.034 (4)	0.052 (4)	0.057 (4)	−0.004 (3)	−0.010 (3)	0.015 (3)
C10	0.030 (4)	0.065 (5)	0.061 (5)	−0.017 (4)	−0.017 (3)	0.009 (4)
C11	0.042 (4)	0.053 (4)	0.058 (4)	−0.019 (4)	−0.015 (3)	0.003 (3)
C12	0.037 (4)	0.029 (3)	0.049 (4)	−0.002 (3)	−0.013 (3)	0.002 (3)
C13	0.099 (4)	0.059 (3)	0.042 (2)	−0.016 (3)	−0.004 (2)	−0.004 (2)
C14	0.099 (4)	0.059 (3)	0.042 (2)	−0.016 (3)	−0.004 (2)	−0.004 (2)
C15	0.099 (4)	0.059 (3)	0.042 (2)	−0.016 (3)	−0.004 (2)	−0.004 (2)
C16	0.017 (3)	0.029 (3)	0.032 (3)	−0.007 (2)	0.001 (2)	−0.002 (2)
C17	0.044 (4)	0.034 (4)	0.039 (4)	−0.010 (3)	−0.009 (3)	−0.002 (3)
C18	0.055 (4)	0.026 (3)	0.054 (4)	−0.009 (3)	−0.015 (3)	0.003 (3)
C19	0.036 (4)	0.038 (4)	0.043 (4)	−0.005 (3)	−0.005 (3)	0.010 (3)
C20	0.050 (4)	0.050 (4)	0.023 (3)	−0.002 (3)	−0.003 (3)	0.002 (3)
C21	0.045 (4)	0.033 (3)	0.033 (3)	−0.002 (3)	−0.001 (3)	−0.005 (3)
C22	0.018 (3)	0.031 (3)	0.033 (3)	−0.003 (2)	−0.003 (2)	−0.010 (2)
C23	0.031 (4)	0.071 (5)	0.050 (4)	−0.023 (4)	0.002 (3)	0.001 (4)
C24	0.027 (4)	0.099 (6)	0.077 (6)	−0.031 (4)	0.004 (4)	−0.012 (5)
C25	0.029 (4)	0.077 (5)	0.073 (5)	−0.013 (4)	−0.013 (4)	−0.037 (4)
C26	0.036 (4)	0.072 (5)	0.056 (4)	−0.008 (4)	−0.019 (3)	−0.024 (4)
C27	0.036 (4)	0.045 (4)	0.036 (4)	−0.010 (3)	−0.008 (3)	−0.015 (3)
C28	0.023 (3)	0.031 (3)	0.027 (3)	−0.009 (2)	−0.007 (2)	−0.007 (2)
C29	0.040 (4)	0.032 (3)	0.033 (3)	−0.008 (3)	−0.014 (3)	0.001 (3)
C30	0.048 (4)	0.029 (3)	0.039 (4)	−0.011 (3)	−0.010 (3)	0.003 (3)
C31	0.025 (3)	0.030 (3)	0.024 (3)	−0.007 (2)	−0.009 (2)	−0.005 (2)
C32	0.034 (3)	0.029 (3)	0.031 (3)	−0.004 (3)	−0.006 (3)	−0.006 (2)
C33	0.030 (3)	0.042 (4)	0.036 (3)	−0.010 (3)	−0.001 (3)	−0.010 (3)
C34	0.022 (3)	0.059 (4)	0.045 (4)	−0.004 (3)	−0.006 (3)	−0.011 (3)
C35	0.038 (4)	0.065 (5)	0.044 (4)	−0.002 (3)	−0.021 (3)	0.006 (3)
C36	0.029 (3)	0.058 (4)	0.031 (3)	−0.013 (3)	−0.008 (3)	0.001 (3)
C37	0.032 (3)	0.033 (3)	0.034 (3)	−0.015 (3)	0.005 (3)	−0.010 (3)
C38	0.058 (4)	0.049 (4)	0.039 (4)	−0.026 (4)	0.000 (3)	−0.009 (3)
C39	0.103 (7)	0.065 (5)	0.043 (4)	−0.047 (5)	0.013 (4)	−0.024 (4)
C40	0.068 (6)	0.037 (5)	0.091 (7)	−0.013 (4)	0.032 (5)	−0.012 (4)
C41	0.047 (5)	0.047 (5)	0.109 (7)	−0.008 (4)	0.005 (5)	−0.004 (5)
C42	0.040 (4)	0.032 (4)	0.068 (5)	−0.006 (3)	−0.008 (3)	0.001 (3)
C43	0.035 (3)	0.036 (3)	0.035 (3)	−0.009 (3)	−0.010 (3)	−0.003 (3)
C44	0.028 (3)	0.045 (4)	0.052 (4)	−0.006 (3)	−0.003 (3)	0.003 (3)
C45	0.037 (4)	0.053 (4)	0.058 (4)	−0.008 (3)	0.006 (3)	0.004 (3)

### Geometric parameters (Å, °)

**Table:**

W1—S4	2.1314 (15)	C17—C18	1.380 (8)
W1—S1	2.2445 (13)	C17—H17	0.9500
W1—S3	2.2483 (13)	C18—C19	1.376 (8)
W1—S2	2.2533 (13)	C18—H18	0.9500
W1—Cu3	2.7033 (7)	C19—C20	1.365 (8)
W1—Cu1	2.7173 (8)	C19—H19	0.9500
W1—Cu2	2.7272 (7)	C20—C21	1.386 (8)
I1—Cu1	2.8056 (8)	C20—H20	0.9500
I1—Cu2	2.9008 (8)	C21—H21	0.9500
Cu1—P1	2.2150 (16)	C22—C27	1.378 (8)
Cu1—S3	2.3094 (15)	C22—C23	1.391 (8)
Cu1—S1	2.3194 (14)	C23—C24	1.385 (9)
Cu2—P2	2.2241 (14)	C23—H23	0.9500
Cu2—S1	2.3015 (15)	C24—C25	1.369 (10)
Cu2—S2	2.3036 (15)	C24—H24	0.9500
Cu3—P3	2.2209 (15)	C25—C26	1.358 (10)
Cu3—S2	2.2833 (15)	C25—H25	0.9500
Cu3—S3	2.2849 (15)	C26—C27	1.386 (8)
P1—C1	1.809 (5)	C26—H26	0.9500
P1—C7	1.809 (6)	C27—H27	0.9500
P1—C13	1.826 (7)	C28—C29	1.522 (7)
P2—C16	1.823 (6)	C28—H28A	0.9900
P2—C22	1.825 (5)	C28—H28B	0.9900
P2—C28	1.835 (5)	C29—C30	1.519 (7)
P3—C37	1.804 (6)	C29—H29A	0.9900
P3—C31	1.821 (5)	C29—H29B	0.9900
P3—C43	1.833 (6)	C30—H30A	0.9800
C1—C2	1.386 (8)	C30—H30B	0.9800
C1—C6	1.397 (7)	C30—H30C	0.9800
C2—C3	1.396 (9)	C31—C36	1.378 (8)
C2—H2	0.9500	C31—C32	1.390 (7)
C3—C4	1.367 (9)	C32—C33	1.385 (8)
C3—H3	0.9500	C32—H32	0.9500
C4—C5	1.362 (9)	C33—C34	1.365 (8)
C4—H4	0.9500	C33—H33	0.9500
C5—C6	1.378 (8)	C34—C35	1.384 (9)
C5—H5	0.9500	C34—H34	0.9500
C6—H6	0.9500	C35—C36	1.387 (8)
C7—C12	1.369 (8)	C35—H35	0.9500
C7—C8	1.413 (8)	C36—H36	0.9500
C8—C9	1.378 (8)	C37—C42	1.385 (9)
C8—H8	0.9500	C37—C38	1.395 (8)
C9—C10	1.380 (9)	C38—C39	1.392 (9)
C9—H9	0.9500	C38—H38	0.9500
C10—C11	1.376 (9)	C39—C40	1.386 (12)
C10—H10	0.9500	C39—H39	0.9500
C11—C12	1.388 (8)	C40—C41	1.337 (12)
C11—H11	0.9500	C40—H40	0.9500
C12—H12	0.9500	C41—C42	1.378 (10)
C13—C14	1.520 (10)	C41—H41	0.9500
C13—H13A	0.9900	C42—H42	0.9500
C13—H13B	0.9900	C43—C44	1.521 (8)
C14—C15	1.458 (9)	C43—H43A	0.9900
C14—H14A	0.9900	C43—H43B	0.9900
C14—H14B	0.9900	C44—C45	1.516 (8)
C15—H15A	0.9800	C44—H44A	0.9900
C15—H15B	0.9800	C44—H44B	0.9900
C15—H15C	0.9800	C45—H45A	0.9800
C16—C17	1.384 (8)	C45—H45B	0.9800
C16—C21	1.392 (8)	C45—H45C	0.9800
			
S4—W1—S1	110.37 (6)	H15A—C15—H15B	109.5
S4—W1—S3	111.01 (6)	C14—C15—H15C	109.5
S1—W1—S3	107.67 (5)	H15A—C15—H15C	109.5
S4—W1—S2	112.73 (6)	H15B—C15—H15C	109.5
S1—W1—S2	107.37 (5)	C17—C16—C21	118.2 (5)
S3—W1—S2	107.48 (5)	C17—C16—P2	118.0 (4)
Cu3—W1—Cu1	73.06 (2)	C21—C16—P2	123.8 (4)
Cu3—W1—Cu2	75.35 (2)	C18—C17—C16	120.7 (6)
Cu1—W1—Cu2	71.69 (2)	C18—C17—H17	119.7
Cu1—I1—Cu2	67.91 (2)	C16—C17—H17	119.7
P1—Cu1—S3	119.19 (6)	C19—C18—C17	120.6 (6)
P1—Cu1—S1	120.30 (6)	C19—C18—H18	119.7
S3—Cu1—S1	103.18 (5)	C17—C18—H18	119.7
P1—Cu1—W1	153.07 (5)	C20—C19—C18	119.3 (6)
S3—Cu1—W1	52.37 (4)	C20—C19—H19	120.3
S1—Cu1—W1	52.20 (4)	C18—C19—H19	120.3
P1—Cu1—I1	106.09 (5)	C19—C20—C21	120.7 (6)
S3—Cu1—I1	105.67 (4)	C19—C20—H20	119.6
S1—Cu1—I1	99.94 (4)	C21—C20—H20	119.6
W1—Cu1—I1	100.82 (2)	C20—C21—C16	120.4 (6)
P2—Cu2—S1	119.38 (6)	C20—C21—H21	119.8
P2—Cu2—S2	121.62 (6)	C16—C21—H21	119.8
S1—Cu2—S2	103.81 (5)	C27—C22—C23	119.3 (5)
P2—Cu2—W1	152.86 (5)	C27—C22—P2	118.5 (4)
S1—Cu2—W1	52.18 (3)	C23—C22—P2	121.7 (5)
S2—Cu2—W1	52.40 (4)	C24—C23—C22	119.4 (7)
P2—Cu2—I1	108.79 (4)	C24—C23—H23	120.3
S1—Cu2—I1	97.69 (4)	C22—C23—H23	120.3
S2—Cu2—I1	101.62 (4)	C25—C24—C23	121.0 (7)
W1—Cu2—I1	98.23 (2)	C25—C24—H24	119.5
P3—Cu3—S2	125.45 (6)	C23—C24—H24	119.5
P3—Cu3—S3	122.84 (6)	C26—C25—C24	119.3 (6)
S2—Cu3—S3	105.23 (5)	C26—C25—H25	120.4
P3—Cu3—W1	163.37 (5)	C24—C25—H25	120.4
S2—Cu3—W1	52.91 (4)	C25—C26—C27	121.2 (7)
S3—Cu3—W1	52.77 (4)	C25—C26—H26	119.4
W1—S1—Cu2	73.71 (4)	C27—C26—H26	119.4
W1—S1—Cu1	73.06 (4)	C22—C27—C26	119.7 (6)
Cu2—S1—Cu1	87.25 (5)	C22—C27—H27	120.1
W1—S2—Cu3	73.15 (4)	C26—C27—H27	120.1
W1—S2—Cu2	73.51 (4)	C29—C28—P2	113.9 (4)
Cu3—S2—Cu2	92.70 (6)	C29—C28—H28A	108.8
W1—S3—Cu3	73.21 (4)	P2—C28—H28A	108.8
W1—S3—Cu1	73.18 (4)	C29—C28—H28B	108.8
Cu3—S3—Cu1	89.22 (5)	P2—C28—H28B	108.8
C1—P1—C7	104.3 (3)	H28A—C28—H28B	107.7
C1—P1—C13	104.6 (3)	C30—C29—C28	112.3 (5)
C7—P1—C13	103.2 (3)	C30—C29—H29A	109.2
C1—P1—Cu1	115.09 (19)	C28—C29—H29A	109.2
C7—P1—Cu1	113.47 (19)	C30—C29—H29B	109.2
C13—P1—Cu1	114.9 (3)	C28—C29—H29B	109.2
C16—P2—C22	104.4 (2)	H29A—C29—H29B	107.9
C16—P2—C28	105.2 (2)	C29—C30—H30A	109.5
C22—P2—C28	101.2 (2)	C29—C30—H30B	109.5
C16—P2—Cu2	114.21 (17)	H30A—C30—H30B	109.5
C22—P2—Cu2	115.37 (18)	C29—C30—H30C	109.5
C28—P2—Cu2	114.94 (17)	H30A—C30—H30C	109.5
C37—P3—C31	103.9 (3)	H30B—C30—H30C	109.5
C37—P3—C43	100.6 (3)	C36—C31—C32	118.6 (5)
C31—P3—C43	105.7 (3)	C36—C31—P3	123.7 (4)
C37—P3—Cu3	115.4 (2)	C32—C31—P3	117.8 (4)
C31—P3—Cu3	115.10 (17)	C33—C32—C31	120.4 (5)
C43—P3—Cu3	114.50 (19)	C33—C32—H32	119.8
C2—C1—C6	117.9 (5)	C31—C32—H32	119.8
C2—C1—P1	123.4 (4)	C34—C33—C32	120.7 (6)
C6—C1—P1	118.7 (4)	C34—C33—H33	119.7
C1—C2—C3	119.9 (6)	C32—C33—H33	119.7
C1—C2—H2	120.0	C33—C34—C35	119.5 (6)
C3—C2—H2	120.0	C33—C34—H34	120.3
C4—C3—C2	120.9 (6)	C35—C34—H34	120.3
C4—C3—H3	119.5	C34—C35—C36	120.1 (6)
C2—C3—H3	119.5	C34—C35—H35	120.0
C5—C4—C3	119.7 (6)	C36—C35—H35	120.0
C5—C4—H4	120.2	C31—C36—C35	120.8 (6)
C3—C4—H4	120.2	C31—C36—H36	119.6
C4—C5—C6	120.4 (6)	C35—C36—H36	119.6
C4—C5—H5	119.8	C42—C37—C38	119.1 (6)
C6—C5—H5	119.8	C42—C37—P3	120.6 (5)
C5—C6—C1	121.2 (6)	C38—C37—P3	120.0 (5)
C5—C6—H6	119.4	C39—C38—C37	119.6 (7)
C1—C6—H6	119.4	C39—C38—H38	120.2
C12—C7—C8	118.3 (5)	C37—C38—H38	120.2
C12—C7—P1	120.8 (5)	C40—C39—C38	119.1 (8)
C8—C7—P1	120.8 (4)	C40—C39—H39	120.4
C9—C8—C7	120.3 (6)	C38—C39—H39	120.4
C9—C8—H8	119.8	C41—C40—C39	121.3 (8)
C7—C8—H8	119.8	C41—C40—H40	119.3
C8—C9—C10	119.8 (6)	C39—C40—H40	119.3
C8—C9—H9	120.1	C40—C41—C42	120.5 (8)
C10—C9—H9	120.1	C40—C41—H41	119.7
C11—C10—C9	120.8 (6)	C42—C41—H41	119.7
C11—C10—H10	119.6	C41—C42—C37	120.3 (7)
C9—C10—H10	119.6	C41—C42—H42	119.9
C10—C11—C12	119.0 (6)	C37—C42—H42	119.9
C10—C11—H11	120.5	C44—C43—P3	112.6 (4)
C12—C11—H11	120.5	C44—C43—H43A	109.1
C7—C12—C11	121.8 (6)	P3—C43—H43A	109.1
C7—C12—H12	119.1	C44—C43—H43B	109.1
C11—C12—H12	119.1	P3—C43—H43B	109.1
C14—C13—P1	112.5 (5)	H43A—C43—H43B	107.8
C14—C13—H13A	109.1	C45—C44—C43	112.7 (5)
P1—C13—H13A	109.1	C45—C44—H44A	109.1
C14—C13—H13B	109.1	C43—C44—H44A	109.1
P1—C13—H13B	109.1	C45—C44—H44B	109.1
H13A—C13—H13B	107.8	C43—C44—H44B	109.1
C15—C14—C13	114.9 (6)	H44A—C44—H44B	107.8
C15—C14—H14A	108.5	C44—C45—H45A	109.5
C13—C14—H14A	108.5	C44—C45—H45B	109.5
C15—C14—H14B	108.5	H45A—C45—H45B	109.5
C13—C14—H14B	108.5	C44—C45—H45C	109.5
H14A—C14—H14B	107.5	H45A—C45—H45C	109.5
C14—C15—H15A	109.5	H45B—C45—H45C	109.5
C14—C15—H15B	109.5		
			
S4—W1—Cu1—P1	−1.55 (13)	S1—Cu1—S3—Cu3	−85.43 (6)
S1—W1—Cu1—P1	−83.29 (11)	W1—Cu1—S3—Cu3	−72.68 (4)
S3—W1—Cu1—P1	80.94 (11)	I1—Cu1—S3—Cu3	19.04 (5)
S2—W1—Cu1—P1	178.85 (11)	S3—Cu1—P1—C1	−54.4 (2)
Cu3—W1—Cu1—P1	138.44 (11)	S1—Cu1—P1—C1	176.6 (2)
Cu2—W1—Cu1—P1	−141.79 (11)	W1—Cu1—P1—C1	−118.1 (2)
S4—W1—Cu1—S3	−82.49 (8)	I1—Cu1—P1—C1	64.4 (2)
S1—W1—Cu1—S3	−164.23 (6)	S3—Cu1—P1—C7	65.6 (2)
S2—W1—Cu1—S3	97.92 (6)	S1—Cu1—P1—C7	−63.4 (2)
Cu3—W1—Cu1—S3	57.51 (5)	W1—Cu1—P1—C7	2.0 (2)
Cu2—W1—Cu1—S3	137.27 (5)	I1—Cu1—P1—C7	−175.5 (2)
S4—W1—Cu1—S1	81.74 (8)	S3—Cu1—P1—C13	−176.0 (3)
S3—W1—Cu1—S1	164.23 (6)	S1—Cu1—P1—C13	55.0 (3)
S2—W1—Cu1—S1	−97.86 (6)	W1—Cu1—P1—C13	120.4 (3)
Cu3—W1—Cu1—S1	−138.27 (5)	I1—Cu1—P1—C13	−57.1 (3)
Cu2—W1—Cu1—S1	−58.50 (4)	S1—Cu2—P2—C16	−38.1 (2)
S4—W1—Cu1—I1	175.99 (6)	S2—Cu2—P2—C16	−170.09 (19)
S1—W1—Cu1—I1	94.25 (5)	W1—Cu2—P2—C16	−101.8 (2)
S3—W1—Cu1—I1	−101.52 (5)	I1—Cu2—P2—C16	72.6 (2)
S2—W1—Cu1—I1	−3.61 (4)	S1—Cu2—P2—C22	−159.1 (2)
Cu3—W1—Cu1—I1	−44.02 (2)	S2—Cu2—P2—C22	68.9 (2)
Cu2—W1—Cu1—I1	35.75 (2)	W1—Cu2—P2—C22	137.2 (2)
Cu2—I1—Cu1—P1	144.60 (5)	I1—Cu2—P2—C22	−48.4 (2)
Cu2—I1—Cu1—S3	−87.95 (4)	S1—Cu2—P2—C28	83.6 (2)
Cu2—I1—Cu1—S1	18.89 (4)	S2—Cu2—P2—C28	−48.3 (2)
Cu2—I1—Cu1—W1	−34.24 (2)	W1—Cu2—P2—C28	20.0 (2)
S4—W1—Cu2—P2	−0.94 (13)	I1—Cu2—P2—C28	−165.68 (19)
S1—W1—Cu2—P2	81.23 (11)	S2—Cu3—P3—C37	−157.25 (19)
S3—W1—Cu2—P2	177.22 (11)	S3—Cu3—P3—C37	55.3 (2)
S2—W1—Cu2—P2	−87.04 (11)	W1—Cu3—P3—C37	124.5 (2)
Cu3—W1—Cu2—P2	−142.85 (11)	S2—Cu3—P3—C31	−36.1 (2)
Cu1—W1—Cu2—P2	140.49 (11)	S3—Cu3—P3—C31	176.4 (2)
S4—W1—Cu2—S1	−82.17 (8)	W1—Cu3—P3—C31	−114.4 (2)
S3—W1—Cu2—S1	95.99 (6)	S2—Cu3—P3—C43	86.6 (2)
S2—W1—Cu2—S1	−168.27 (6)	S3—Cu3—P3—C43	−60.8 (2)
Cu3—W1—Cu2—S1	135.92 (5)	W1—Cu3—P3—C43	8.4 (3)
Cu1—W1—Cu2—S1	59.26 (4)	C7—P1—C1—C2	131.6 (6)
S4—W1—Cu2—S2	86.10 (8)	C13—P1—C1—C2	23.6 (7)
S1—W1—Cu2—S2	168.27 (6)	Cu1—P1—C1—C2	−103.4 (5)
S3—W1—Cu2—S2	−95.74 (6)	C7—P1—C1—C6	−49.4 (5)
Cu3—W1—Cu2—S2	−55.81 (5)	C13—P1—C1—C6	−157.5 (5)
Cu1—W1—Cu2—S2	−132.47 (5)	Cu1—P1—C1—C6	75.5 (5)
S4—W1—Cu2—I1	−175.54 (7)	C6—C1—C2—C3	0.6 (10)
S1—W1—Cu2—I1	−93.37 (5)	P1—C1—C2—C3	179.6 (6)
S3—W1—Cu2—I1	2.62 (5)	C1—C2—C3—C4	0.3 (12)
S2—W1—Cu2—I1	98.36 (5)	C2—C3—C4—C5	−1.0 (12)
Cu3—W1—Cu2—I1	42.55 (2)	C3—C4—C5—C6	0.7 (11)
Cu1—W1—Cu2—I1	−34.11 (2)	C4—C5—C6—C1	0.2 (10)
Cu1—I1—Cu2—P2	−143.59 (5)	C2—C1—C6—C5	−0.9 (9)
Cu1—I1—Cu2—S1	−18.92 (4)	P1—C1—C6—C5	−179.9 (5)
Cu1—I1—Cu2—S2	86.96 (4)	C1—P1—C7—C12	142.3 (5)
Cu1—I1—Cu2—W1	33.81 (2)	C13—P1—C7—C12	−108.6 (5)
S4—W1—Cu3—P3	3.64 (19)	Cu1—P1—C7—C12	16.3 (5)
S1—W1—Cu3—P3	−175.22 (17)	C1—P1—C7—C8	−41.2 (5)
S3—W1—Cu3—P3	−80.55 (17)	C13—P1—C7—C8	67.9 (5)
S2—W1—Cu3—P3	90.54 (17)	Cu1—P1—C7—C8	−167.2 (4)
Cu1—W1—Cu3—P3	−138.54 (17)	C12—C7—C8—C9	−0.7 (9)
Cu2—W1—Cu3—P3	146.52 (17)	P1—C7—C8—C9	−177.3 (5)
S4—W1—Cu3—S2	−86.90 (8)	C7—C8—C9—C10	0.9 (9)
S1—W1—Cu3—S2	94.24 (6)	C8—C9—C10—C11	−0.9 (10)
S3—W1—Cu3—S2	−171.09 (7)	C9—C10—C11—C12	0.8 (10)
Cu1—W1—Cu3—S2	130.92 (5)	C8—C7—C12—C11	0.6 (9)
Cu2—W1—Cu3—S2	55.97 (5)	P1—C7—C12—C11	177.2 (5)
S4—W1—Cu3—S3	84.19 (8)	C10—C11—C12—C7	−0.6 (10)
S1—W1—Cu3—S3	−94.67 (6)	C1—P1—C13—C14	176.6 (6)
S2—W1—Cu3—S3	171.09 (7)	C7—P1—C13—C14	67.8 (7)
Cu1—W1—Cu3—S3	−57.99 (5)	Cu1—P1—C13—C14	−56.3 (7)
Cu2—W1—Cu3—S3	−132.93 (5)	P1—C13—C14—C15	−167.9 (6)
S4—W1—S1—Cu2	133.16 (5)	C22—P2—C16—C17	79.5 (5)
S3—W1—S1—Cu2	−105.53 (5)	C28—P2—C16—C17	−174.4 (4)
S2—W1—S1—Cu2	9.93 (5)	Cu2—P2—C16—C17	−47.4 (5)
Cu3—W1—S1—Cu2	−47.70 (4)	C22—P2—C16—C21	−100.2 (5)
Cu1—W1—S1—Cu2	−92.11 (4)	C28—P2—C16—C21	5.9 (5)
S4—W1—S1—Cu1	−134.73 (5)	Cu2—P2—C16—C21	132.9 (4)
S3—W1—S1—Cu1	−13.42 (5)	C21—C16—C17—C18	1.2 (9)
S2—W1—S1—Cu1	102.04 (5)	P2—C16—C17—C18	−178.6 (5)
Cu3—W1—S1—Cu1	44.41 (4)	C16—C17—C18—C19	−0.9 (10)
Cu2—W1—S1—Cu1	92.11 (4)	C17—C18—C19—C20	0.3 (10)
P2—Cu2—S1—W1	−148.84 (6)	C18—C19—C20—C21	−0.1 (10)
S2—Cu2—S1—W1	−9.55 (5)	C19—C20—C21—C16	0.4 (10)
I1—Cu2—S1—W1	94.48 (3)	C17—C16—C21—C20	−0.9 (9)
P2—Cu2—S1—Cu1	138.01 (6)	P2—C16—C21—C20	178.8 (5)
S2—Cu2—S1—Cu1	−82.69 (6)	C16—P2—C22—C27	−161.2 (4)
W1—Cu2—S1—Cu1	−73.15 (4)	C28—P2—C22—C27	89.7 (5)
I1—Cu2—S1—Cu1	21.33 (4)	Cu2—P2—C22—C27	−35.0 (5)
P1—Cu1—S1—W1	148.60 (6)	C16—P2—C22—C23	26.4 (6)
S3—Cu1—S1—W1	12.78 (5)	C28—P2—C22—C23	−82.7 (5)
I1—Cu1—S1—W1	−96.05 (3)	Cu2—P2—C22—C23	152.5 (5)
P1—Cu1—S1—Cu2	−137.59 (6)	C27—C22—C23—C24	0.0 (10)
S3—Cu1—S1—Cu2	86.58 (5)	P2—C22—C23—C24	172.4 (5)
W1—Cu1—S1—Cu2	73.81 (4)	C22—C23—C24—C25	−1.7 (11)
I1—Cu1—S1—Cu2	−22.24 (4)	C23—C24—C25—C26	2.2 (12)
S4—W1—S2—Cu3	130.20 (5)	C24—C25—C26—C27	−1.0 (11)
S1—W1—S2—Cu3	−108.04 (5)	C23—C22—C27—C26	1.1 (9)
S3—W1—S2—Cu3	7.55 (6)	P2—C22—C27—C26	−171.5 (5)
Cu1—W1—S2—Cu3	−50.10 (4)	C25—C26—C27—C22	−0.6 (10)
Cu2—W1—S2—Cu3	−98.11 (5)	C16—P2—C28—C29	−176.6 (4)
S4—W1—S2—Cu2	−131.70 (5)	C22—P2—C28—C29	−68.1 (4)
S1—W1—S2—Cu2	−9.93 (5)	Cu2—P2—C28—C29	56.9 (4)
S3—W1—S2—Cu2	105.66 (5)	P2—C28—C29—C30	180.0 (4)
Cu3—W1—S2—Cu2	98.11 (5)	C37—P3—C31—C36	−101.5 (5)
Cu1—W1—S2—Cu2	48.01 (4)	C43—P3—C31—C36	3.9 (6)
P3—Cu3—S2—W1	−159.43 (6)	Cu3—P3—C31—C36	131.3 (4)
S3—Cu3—S2—W1	−7.34 (5)	C37—P3—C31—C32	77.4 (5)
P3—Cu3—S2—Cu2	128.70 (7)	C43—P3—C31—C32	−177.1 (4)
S3—Cu3—S2—Cu2	−79.21 (6)	Cu3—P3—C31—C32	−49.7 (5)
W1—Cu3—S2—Cu2	−71.87 (4)	C36—C31—C32—C33	2.1 (8)
P2—Cu2—S2—W1	147.65 (6)	P3—C31—C32—C33	−176.9 (4)
S1—Cu2—S2—W1	9.52 (5)	C31—C32—C33—C34	−2.8 (9)
I1—Cu2—S2—W1	−91.50 (3)	C32—C33—C34—C35	1.4 (9)
P2—Cu2—S2—Cu3	−140.81 (6)	C33—C34—C35—C36	0.5 (10)
S1—Cu2—S2—Cu3	81.06 (6)	C32—C31—C36—C35	−0.1 (9)
W1—Cu2—S2—Cu3	71.54 (4)	P3—C31—C36—C35	178.8 (5)
I1—Cu2—S2—Cu3	−19.96 (5)	C34—C35—C36—C31	−1.2 (10)
S4—W1—S3—Cu3	−131.24 (6)	C31—P3—C37—C42	−143.2 (5)
S1—W1—S3—Cu3	107.85 (5)	C43—P3—C37—C42	107.6 (5)
S2—W1—S3—Cu3	−7.54 (6)	Cu3—P3—C37—C42	−16.2 (5)
Cu1—W1—S3—Cu3	94.37 (5)	C31—P3—C37—C38	42.7 (5)
Cu2—W1—S3—Cu3	50.12 (5)	C43—P3—C37—C38	−66.5 (5)
S4—W1—S3—Cu1	134.38 (6)	Cu3—P3—C37—C38	169.7 (4)
S1—W1—S3—Cu1	13.47 (5)	C42—C37—C38—C39	−1.6 (9)
S2—W1—S3—Cu1	−101.92 (5)	P3—C37—C38—C39	172.6 (5)
Cu3—W1—S3—Cu1	−94.37 (5)	C37—C38—C39—C40	0.3 (10)
Cu2—W1—S3—Cu1	−44.26 (4)	C38—C39—C40—C41	0.7 (11)
P3—Cu3—S3—W1	160.37 (6)	C39—C40—C41—C42	−0.4 (12)
S2—Cu3—S3—W1	7.36 (5)	C40—C41—C42—C37	−1.0 (11)
P3—Cu3—S3—Cu1	−126.98 (6)	C38—C37—C42—C41	2.0 (9)
S2—Cu3—S3—Cu1	80.01 (6)	P3—C37—C42—C41	−172.2 (5)
W1—Cu3—S3—Cu1	72.65 (4)	C37—P3—C43—C44	−65.6 (5)
P1—Cu1—S3—W1	−149.18 (6)	C31—P3—C43—C44	−173.5 (4)
S1—Cu1—S3—W1	−12.75 (5)	Cu3—P3—C43—C44	58.8 (5)
I1—Cu1—S3—W1	91.72 (3)	P3—C43—C44—C45	176.9 (4)
P1—Cu1—S3—Cu3	138.14 (6)		

### Hydrogen-bond geometry (Å, °)

**Table:**

*D*—H···*A*	*D*—H	H···*A*	*D*···*A*	*D*—H···*A*
C10—H10···I1^i^	0.95	3.14	3.936 (7)	142

Symmetry codes: (i) *x*+1, *y*, *z*.

## References

[bb1] Hou, H. W., Liang, B., Xin, X., Yu, K., Ge, P., Ji, W. & Shi, S. (1996). *J. Chem. Soc. Faraday Trans.* **92**, 2343–2346.

[bb2] Hou, H. W., Xin, X. Q. & Shi, S. (1996). *Coord. Chem. Rev.* **153**, 25–56.

[bb3] Müller, A., Diemann, E., Jostes, R. & Bögge, H. (1981). *Angew. Chem. Int. Ed. Engl.* **20**, 934–955.

[bb4] Niu, Y. Y., Zheng, H. G., Hou, H. W. & Xin, X. Q. (2004). *Coord. Chem. Rev.* **248**, 169–183.

[bb5] Rigaku (2000). *CrystalClear *and *CrystalStructure* Rigaku Corporation, Tokyo, Japan.

[bb6] Sheldrick, G. M. (1996). *SADABS* University of Göttingen, Germany.

[bb7] Sheldrick, G. M. (2008). *Acta Cryst.* A**64**, 112–122. 10.1107/S0108767307043930 18156677

[bb8] Zhang, C., Song, Y. L. & Wang, X. (2007). *Coord. Chem. Rev.* **251**, 111–141.

